# First Case of Legionnaire's Disease Caused by *Legionella anisa* in Spain and the Limitations on the Diagnosis of *Legionella* non-*pneumophila* Infections

**DOI:** 10.1371/journal.pone.0159726

**Published:** 2016-07-21

**Authors:** Lucianna Vaccaro, Fernando Izquierdo, Angela Magnet, Carolina Hurtado, Mireya A. Salinas, Thiago Santos Gomes, Santiago Angulo, Santiago Salso, Jesús Pelaez, Maria Isabel Tejeda, Almudena Alhambra, Carmen Gómez, Ana Enríquez, Eva Estirado, Soledad Fenoy, Carmen del Aguila

**Affiliations:** 1 Departamento de Ciencias Farmacéuticas y de la Salud, Facultad de Farmacia, Universidad San Pablo CEU, Alcorcón, Madrid, Spain; 2 CAPES Foundation, Ministry of Education of Brazil, Brasília, Brazil; 3 Hospital Universitario HM Monteprincipe, Boadilla del Monte, Madrid, Spain; 4 Hospital Universitario HM Sanchinarro, Madrid, Madrid, Spain; 5 Hospital Universitario Carlos III, Madrid, Madrid, Spain; University of Louisville, UNITED STATES

## Abstract

Legionnaires’ disease is a severe form of pneumonia, with worldwide relevance, caused by *Legionella* spp. Approximately 90% of all cases of legionellosis are caused by *Legionella pneumophila*, but other species can also be responsible for this infection. These bacteria are transmitted by inhalation of aerosols or aspiration of contaminated water. In Spain, environmental studies have demonstrated the presence of *Legionella* non-*pneumophila* species in drinking water treatment plants and water distribution networks. Aware that this evidence indicates a risk factor and the lack of routine assays designed to detect simultaneously diverse *Legionella* species, we analyzed 210 urine samples from patients presenting clinical manifestations of pneumonia using a semi-nested PCR for partial amplification of the 16S rDNA gene of *Legionella* and a diagnostic method used in hospitals for *Legionella* antigen detection. In this study, we detected a total of 15 cases of legionellosis (7.1%) and the first case of Legionnaires’ disease caused by *L*. *anisa* in Spain. While the conventional method used in hospitals could only detect four cases (1.9%) produced by *L*. *pneumophila* serogroup 1, using PCR, the following species were identified: *Legionella* spp. (10/15), *L*. *pneumophila* (4/15) and *L*. *anisa* (1/15). These results suggest the need to change hospital diagnostic strategies regarding the identification of *Legionella* species associated with this disease. Therefore, the detection of *Legionella* DNA by PCR in urine samples seems to be a suitable alternative method for a sensitive, accurate and rapid diagnosis of *Legionella* pneumonia, caused by *L*. *pneumophila* and also for *L*. non-*pneumophila* species.

## Introduction

*Legionella* spp. are environmental gram-negative bacteria that have been described as causative agents of Legionnaires’ disease. The infection is transmitted by inhalation of aerosols generated from man-made water systems or aspiration of water containing *Legionella* [1, 2]. Legionnaires’ disease can be a severe pneumonia that may be accompanied by systemic symptoms such as fever, diarrhea, myalgia, and impaired renal and liver functions. *Legionella* spp. are also associated with cases of Pontiac fever, which is a self-limiting and mild illness of short duration, without pneumonia [3].

Most cases of legionellosis are community-acquired, followed by travel-associated and nosocomial pneumonia. The highest numbers of cases occur in older people (74–90% of patients ≥50 years) and predominantly in men [4, 5]. The case–fatality rate depends on the severity of the disease; it can reach values greater than 40% in cases of healthcare-associated pneumonia. Thus, recognizing outbreaks and making an early diagnosis are crucial measures for the management of patients with legionellosis, principally in immunocompromised people [6].

Between 2011 and 2012, *Legionella* spp. was the most frequently reported etiological agent (66%) among drinking water–associated waterborne disease outbreaks in the United States and was the only one responsible for all outbreak deaths [7]. The incidence of legionellosis in the United States increased from 3688 confirmed cases in 2012 to 4954 confirmed cases in 2013, according to the latest reports published by the Centers for Disease Control and Prevention (CDC) [8, 9]. In Europe, Legionnaires’ disease is also an important cause of pneumonia, presenting similar figures to those found in the United States. In 2013, the European Centers for Disease Control and Prevention (ECDC) notified 5790 cases of Legionnaires’ disease in Europe with a 10% mortality rate. Italy and France presented the first and second highest prevalence rate in Europe with 23.2 and 21.8%, respectively; followed by Spain with 14% of positive cases [4].

The most common etiological agent of legionellosis worldwide is *L*. *pneumophila* serogroup 1. Also, other *L*. *pneumophila* serogroups and different *Legionella* species (mostly *L*. *longbeachae*, *L*. *bozemanii*, *L*. *dumoffii* and *L*. *micdadei*) have been found to be responsible for Legionnaires’ disease and outbreaks of Pontiac fever, including extrapulmonary infections such as cellulitis, endocarditis and cutaneous infection [10–20]. However, cases by *L*. non*-pneumophila* species are usually under-reported due to the absence of diagnostic methods in hospitals to identify other species than *L*. *pneumophila* [3, 21].

The gold standard method to isolate *Legionella* spp. is culturing on selective medium, but its use has declined because it is time-consuming due to the slow growth of this organism and, also on account of its poor sensitivity, which depends on the severity of the disease and *Legionella* species. Other techniques used for confirmation of cases of legionellosis are: i) a significant rise of *Legionella* antibody levels in serum samples and ii) antigen detection by direct fluorescent antibody (DFA) staining in respiratory secretions and tissue samples. Nevertheless, the use of these methods has also decreased due to false positive results caused by cross-reactions with bacteria and yeast [5, 22].

Currently, most countries use urinary antigen detection as a routine diagnostic method for cases of legionellosis. However, false negative results could occur with these commercial kits, since the majority of them do not allow for the detection of *L*. *pneumophila* non-serogroup 1 and other *Legionella* species [5, 23]. On the other hand, *Legionella* DNA detection through PCR (Polymerase Chain Reaction) can be a potential tool, providing results within a short time period and detecting infections caused by all *Legionella* species and serogroups, with high sensitivity and specificity (>90%) [22]. In fact, the use of PCR for diagnosis of Legionnaires’ disease has continuously increased in recent years, such is the case in Denmark, where several laboratories use PCR as a routine diagnostic method for *Legionella* detection [21]. For 2013, 70% cases of legionellosis in Estonia were diagnosed through PCR, followed by 36% in Denmark and around 21–24% in United Kingdom, Norway and Sweden [4].

Molecular methods have also allowed to carry out several epidemiological surveys that have revealed the presence of pathogenic *L*. non-*pneumophila* species in environmental samples, which represents a risk factor for *Legionella* infections [2, 24, 25]. In Spain, *L*. *feeleii*, *L*. *anisa*, *L*. *donaldsonii*, *L*. *bozemanii*, *L*. *dumoffi* and *L*. *jordanis* have been found in drinking water treatment plants, water distribution networks and cooling towers [26–29]. These *L*. non-*pneumophila* species have been previously described as etiological agents of respiratory tract infections [11, 14, 15]. Considering these evidences and the lack of routine diagnostic methods in hospitals to detect all *L*. *pneumophila* serogroups and *L*. non-*pneumophila* species responsible for cases of legionellosis, we describe in this study the application of a PCR protocol to identify diverse *Legionella* species in urine samples from patients with respiratory symptoms. In addition, all samples were analyzed by one of the most common routine techniques for diagnosis of *Legionella*.

## Materials and Methods

### Samples collection

Between September 2013 and December 2014, a total of 210 urine samples were obtained from patients who attended hospital presenting clinical manifestations of respiratory diseases. The selection of the patients was supported on a combination of signs and symptoms associated with lower respiratory tract infection, according to the guidelines of the Spanish Society of Chest Diseases and Thoracic Surgery (SEPAR) [30]. The main criteria were fever (>38°C), cough, shaking chills, expectoration, chest pain and dyspnea. Samples were collected at Hospital Carlos III, Hospital Universitario HM Sanchinarro and Hospital Universitario HM Monteprincipe (Madrid, Spain).

### Ethics Statement

This epidemiological survey was carried out in compliance with fundamental ethical principles, including those reflected in the Charter of Fundamental Rights of the European Union and the European Convention on Human Rights and its Supplementary Protocols. All participants attested their involvement in this clinical research by means of a written informed consent, which was evaluated and approved by the Research Ethics Committee of the University San Pablo CEU, in accordance with the recommendations of the Spanish Bioethics Committee, the Spanish legislation on Biomedical Research (Law 14/2007, of July 3^rd^) and Personal Data Protection (Organic Law 15/1999 and Royal Decree 1720/2007). These laws define that access to the clinical record for judicial, epidemiological, public health, research or educational purposes carry an obligation to keep the patient’s personal identification data separated from clinical and healthcare data, so that as a rule anonymity is ensured.

### Detection of *Legionella* by urinary antigen

All samples were analyzed with *Legionella* Urinary Antigen Card (Alere BinaxNOW^®^, United States) according to the manufacturer’s instructions. This commercial kit, used in hospitals, is an immunochromatography test for the qualitative detection of *L*. *pneumophila* serogroup 1 antigen [31].

### Genomic DNA extraction

Genomic DNA was extracted from 4.5 mL of urine sample using NucleoSpin^®^ Tissue kit (Macherey-Nagel, Germany) following the manufacturer’s instructions, with a previous 10 minute incubation step at 40°C to dissolve the precipitates from the samples. The extracted DNA was stored at -80°C until PCR analysis.

### PCR and DNA sequence analysis

A semi-nested PCR described by Miyamoto *et al*. [32] was used for partial amplification of the 16S rDNA gene of *Legionella*, with some modifications performed by Magnet *et al*. [26]. The primers used in the first-step of the semi-nested PCR were LEG225 5′-AAGATTAGCCTGCGTCCGAT-3′ and LEG858 5′-GTCAACTTATCGCGTTTGCT-3′ [32]. The amplified products from positive samples in this first-step of PCR were purified using NucleoSpin^®^ Gel and PCR Clean-up (Macherey-Nagel, Germany). These PCR products were then sequenced in both directions by Macrogen laboratories sequencing service (Seoul, Korea). The sequences were analyzed using Bioedit Sequence Alignment Editor 7.0.5.3.

In addition, as negative samples in the first-step of the semi-nested PCR could contain a low concentration of DNA, a second reaction PCR was carried out with internal specific primers (LEG448 5′-GAGGGTTGATAGGTTAAGAGC-3′ and LEG858) to detect *Legionella* spp. (S1 Fig) [32]. Total genomic DNA from *L*. *pneumophila* serogroup 1 (NCTC 12821) and *L*. *feeleii* (Bacteria collection of the University San Pablo CEU) were used as positive controls and elution buffer from the DNA extraction kit as a negative control.

### Data collection

For positive cases, the patients’ clinical history was revised thoroughtly for informational purpose to set up a suitable correlation with our results. Laboratory analysis and image studies were checked as well as the presence of risk factors (chronic lung diseases, immunosuppression, diabetes *mellitus* and exposure to possible source of *Legionella*), signs and symptoms, clinical suspicions of atypical pneumonia, diagnosis of other pathogens and treatments.

### Statistical Analysis

All statistical analyses were performed using the IBM^®^ SPSS Statistics 20 software (Chicago, IL, USA). The results obtained by immunochromatography and PCR were analyzed using McNemar test. *p* < 0.05 was considered to indicate statistical significance.

## Results

### Detection of *Legionella* infections

A total of 210 urine samples from patients with a suspicion of pneumonia were analyzed using an immunochromatographic assay and molecular techniques to detect *Legionella* antigen and DNA, respectively (S1 Fig). The first method (urinary antigen test) proved 4 samples of 210 (1.9%) positive for *L*. *pneumophila* serogroup 1. Regarding the semi-nested PCR, 15 samples of 210 (7.1%) were positive for legionellosis with the following distribution: 4 cases were due to *L*. *pneumophila* (the same cases identified by immunochromatography), 1 case attributed to *L*. *anisa* and in the rest of positive samples (10 cases) the species of *Legionella* was not possible to identify (Table 1). These results show a significant difference (*p* < 0.05) between PCR and immunochromatography for the identification of *Legionella* in urine samples.

**Table 1:** *Legionella* species detected in this study through urinary antigen test and molecular methods.

Bacterium	Immunochromathography	PCR	N° (%) of detected cases
***Legionella spp*.**	0/210	10/210	10/210 (4.7)
***L*. *pneumophila***	4/210	4/210	4/210 (1.9)
***L*. *anisa***	0/210	1/210	1/210 (0.5)
**TOTAL**	4/210 (1.9%)	15/210 (7.1%)	**15/210 (7.1%)**

**Fig 1:**
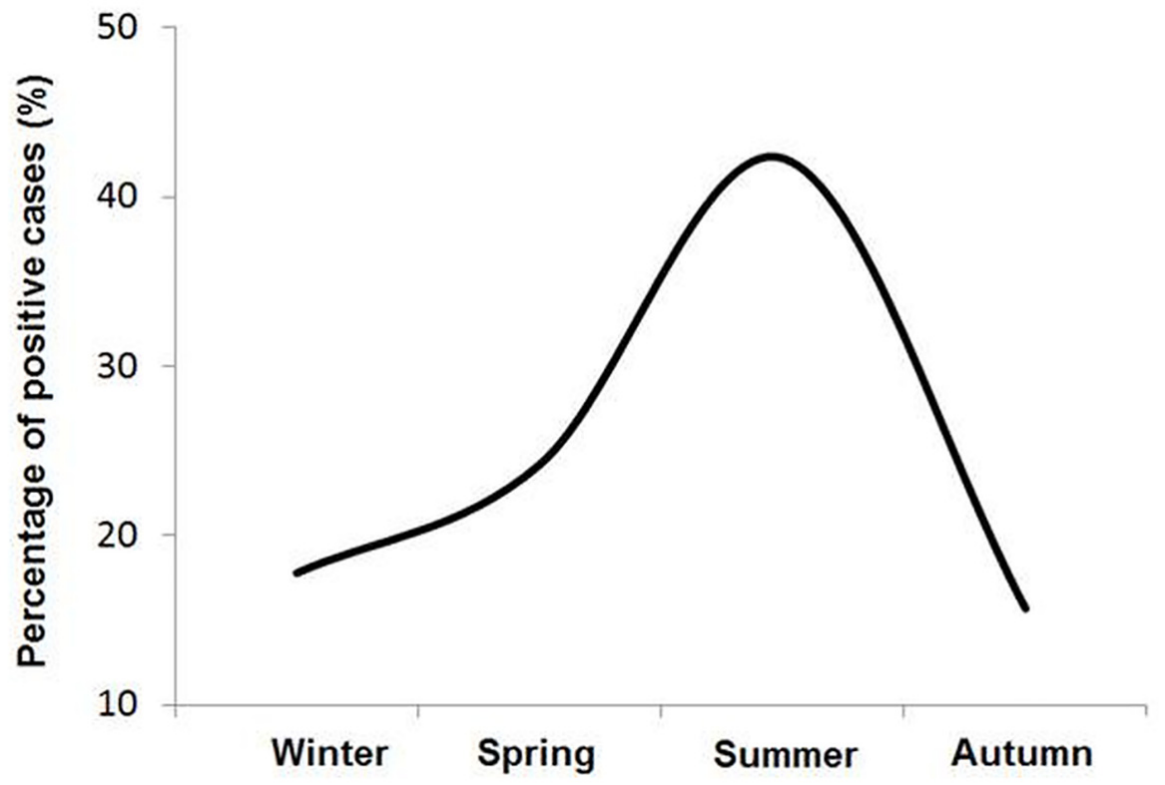
Seasonality of cases of Legionnaires’ disease detected in Madrid between September 2013 and December 2014. The highest peak of cases of legionellosis was observed in summer.

Most of the cases were reported in patients over 50 years of age and in men (Table 2). The main symptoms and signs were fever, hypoxemia, cough and dyspnea. About the image study, 11 of the 15 patients with legionellosis presented infiltrates in the chest X-ray. Radiological signs do not allow to identify the causative agent but the presence of infiltrates coupled with clinical manifestations are considered a gold standard for diagnosing of pneumonia [30]. Regarding the distribution of cases of Legionnaires’ disease by seasonality, a peak was observed during warmer seasons, which is associated with an optimal growth temperature for the rapid multiplication and transmission of *Legionella* though water (Fig 1).

**Table 2:** Distribution of positive cases for *Legionella* classified by gender, age, clinical suspicious of atypical pneumonia and the presence of infiltrate in chest X-rays (n = 15).

CATEGORY	N° positive cases	Percentage (%)
**GENDER**		
Female	5/15	33.3
Male	10/15	66.7
**AGE**		
< 50	6/15	40.0
≥ 50	9/15	60.0
**INFILTRATE (S) IN THE CHEST X-RAY**		
Yes	11/15	73.3
No	4/15	26.7
ATYPICAL PNEUMONIA*		
Yes	11/15	73.3
No	4/15	26.7

*Clinical suspicious of atypical pneumonia. *Note*: Overlapping values between the presence of infiltrates and suspicion of atypical pneumonia does not mean that these correspond exactly to each other.

Furthermore, the case–fatality rate was 7% (1 out of 15 positive cases), similar to the values notified by the ECDC [4]. The reported death corresponded to a 68-year old immunosuppressed male, who suffered acute respiratory distress syndrome. This patient was a confirmed case of Legionnaires’ disease caused by *L*. *pneumophila*, according to clinical and laboratory criteria defined by the European Legionnaires’ Disease Surveillance Network [4]. The etiological agent was detected by inmmunochromatography method and PCR in urine.

### Description of a case of Legionnaires’ disease by *L*. *anisa*

**Fig 2:**
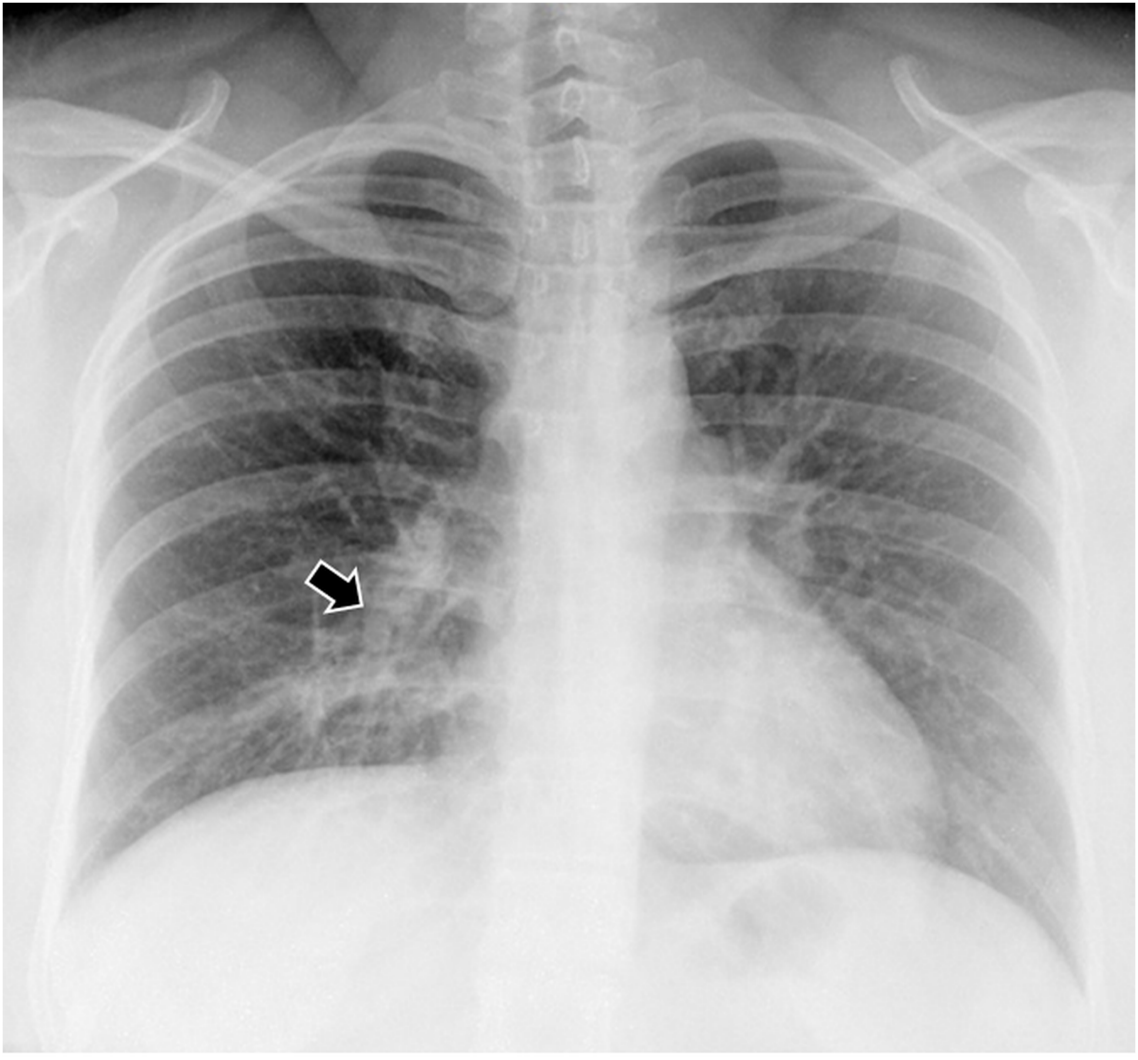
Chest X-ray at admission of a patient with pneumonia by *L*. *anisa*. Radiological signs showed an infiltrate to right lung base (arrow) and a bilateral interstitial pattern.

A case of legionellosis caused by *L*. *anisa* was detected in our study. The patient was a 36 year-old female, immunocompetent, who attended the emergency room of a hospital in Madrid (Spain) presenting a 2-day fever (38.5–39°C), dyspnea, headache and cough. On physical examination, pulmonary auscultation revealed overall decreased breath sounds with discrete expiratory wheezing and bibasilar crackles. Chest X-ray showed an infiltrate to right lung base, which is characteristic for cases of atypical pneumonia (Fig 2).

Laboratory analysis revealed a white blood cell count of 14650/μL with 77% of neutrophil granulocytes, an elevated C-reactive protein level of 47.53 mg/L (reference value <5 mg/L), a high LDH level of 660 U/L (reference value 208–378 U/L) and a condition of hypoxemia (PaO_2_ 57 mmHg). Based on these results, this patient was admitted with a diagnosis of bilateral pneumonia. Samples were collected for microbiological analysis and treatment was started with bronchodilators and levofloxacin (500 mg/day). The following day, ceftriaxone was added to the intravenous antimicrobial regimen (2 g/day).

**Fig 3:**
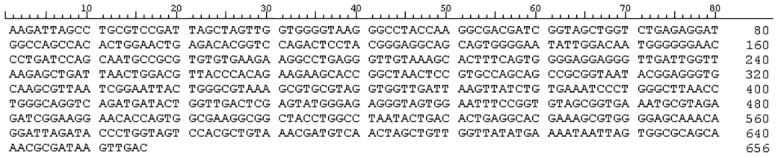
Sequence of amplified product through semi-nested PCR in urine sample from a patient with pneumonia (Accession number KU979014). The analysis of this sequence showed a 100% of homology with *L*. *anisa*.

Regarding microbiological results, respiratory saprophytic microorganisms were isolated from sputum culture. Blood culture was negative as it was the urinary antigen test for the detection of *Streptococcus pneumoniae* and *L*. *pneumophila* serogroup 1. Semi-nested PCR for amplification of *Legionella* DNA was positive in the urine sample. The amplified product of 656 pb was sequenced and a BLAST test was carried out revealing a 100% similarity with gene bank accession number AY744776 that corresponds to *L*. *anisa* (Fig 3).

Three days post-admission, the patient responded to the treatment, presenting an improved clinical condition. The patient was kept on oral antibiotic therapy at home (levofloxacin 500 mg/day and cefixime 400 mg/12 h) during 10 days. After 15 days, examination showed resolution of the infection, with no infiltrates on a chest X-ray.

## Discussion

In Europe, Spain is one of the countries that annually report an elevated number of legionellosis cases, ranking it in third position with 811 cases in 2013. Also, six of the largest European outbreaks of Legionnaires’ disease during the period 2008–2013, took place in Spain. These were associated with various sources including cooling towers, decorative fountains, water systems and pools [4].

In this study, we have detected 7.1% of positive cases of legionellosis (15 out of 210 samples) in patients with pneumonia who attended hospitals in Madrid, Spain. The diagnosis was carried out through routine and molecular techniques in urine samples. Cases of infection by *L*. *pneumophila* (1.9%; n = 4) were identified by both methods, while in the remaining eleven diagnosed cases, *Legionella* spp. (4.7%; n = 10) and *L*. *anisa* (0.5%; n = 1), the detection was only possible through PCR. This difference could be due to the higher sensitivity and specificity of PCR methodology, as well as the existence of cases of *L*. non*-pneumophila* infections not identified by routine assays used in hospitals.

Nowadays, diagnosis of legionellosis is mainly performed by antigen detection tests in urine specifically designed for *L*. *pneumophila* serogroup 1. In 2013, 92% of cases of legionellosis in Spain were diagnosed using this method, while 6% were by isolation in culture and the rest detected through a fourfold titre rise of *Legionella* antibody levels in serum [4]. The detection of *Legionella* antigen in urine is the most common method used to confirm cases of Legionnaires' disease, because it is a rapid, easy, sensitive and specific technique [33, 34]. However, it has one major disadvantage as it does not allow the detection of other *L*. *pneumophila* serogroups or other *Legionella* species related to Legionnaire's disease [23, 35]. This limitation suggests that various cases of legionellosis may not be diagnosed due to lack of tools that can detect, quickly and simultaneously, diverse *Legionella* species in hospitals [21].

For instance, we have reported the first case of Legionnaires’ disease caused by *L*. *anisa* in Spain, which was not detected by the hospital’s conventional method; on the contrary, it was identified through PCR and confirmed by DNA sequencing in urine and sputum samples. This result shows the important role that molecular techniques can have in the diagnosis of legionellosis, which have proved to be more sensitive and specific for the identification of different *Legionella* species such as *L*. *anisa* [18, 36, 37].

*L*. *anisa* is the most frequent *L*. non-*pneumophila* species in the environment, commonly being isolated in cooling towers, drinking water, wastewater treatment plants and in hospital water distribution systems [25, 38–41]. Recently, an environmental survey of state owned water systems revealed the presence of *L*. *anisa* in Midwest and Northeast Spain; it could therefore be associated with the appearance of cases of legionellosis by this species of *Legionella* in Spain [42].

The role of *L*. *anisa* as a causative agent of Legionnaire's disease and outbreaks of Pontiac fever have been previously demonstrated in Australia, France, the United Kingdom, the United States and Japan (S2 Table) [12, 18, 43–49]. Also, this bacterium has been found to produce extrapulmonary infection [18, 50]. Between 2007 and 2008, 19 cases of pneumonia by *L*. non-*pneumophila* species were reported in Europe, 10% caused by *L*. *anisa* (n = 2) [51].

According to the criteria established by the European Legionnaires’ Disease Surveillance Network and the evidences of the association of this microorganism with human disease, the identification of only *L*. *anisa* in our patient with a diagnosis of pneumonia supported by clinical features and imaging study, allowed us to conclude that it could be a case of Legionnaire's disease attributed to *L*. *anisa* [4].

Legionnaires’ disease is not clinically distinguishable from other types of pneumonia, thus the development of powerful tools for the identification of legionellosis and a rational approach to diagnosis are required. Additionally, regular checks for *Legionella* spp. in man-made water systems may be important in preventing cases of Legionnaires’ disease.

## Conclusions

The prevalence of pneumonia caused by *Legionella* in patients with clinical manifestations of respiratory disease was 7.1% in Madrid, in the period from September 2013 to December 2014, with one case a fatal outcome. In this study, we have described the first case of Legionnaire's disease caused by *L*. *anisa* in Spain, which was only detected through PCR and confirmed by DNA sequencing. These molecular methods demonstrated to be more suitable for the detection of cases of legionellosis than the diagnostic test used in hospitals. For this reason, semi-nested PCR for amplification of the 16S rDNA gene of *Legionella* could be a promising method for detection of cases of legionellosis by *L*. *pneumophila* as well as by *L*. non-*pneumophila*. The development of new easy-to-use performance diagnostic tools for simultaneous identification of different *Legionella* species remains an important goal in order to improve the management of patients with *Legionella* infection, allowing an earlier diagnosis so as to select a specific antimicrobial therapy.

## Supporting Information

S1 Fig Workflow scheme followed for detection and characterization of *Legionella* from urine samples. (TIF)

S1 Table The largest outbreaks of Legionnaires’ disease from 1980 to 2015 in Spain. (PDF)

S2 Table Cases of legionellosis attributed to *Legionella anisa* worldwide. (PDF)
