# Correction: First Case of Legionnaire's Disease Caused by *Legionella anisa* in Spain and the Limitations on the Diagnosis of *Legionella* non-*pneumophila* Infections

**DOI:** 10.1371/journal.pone.0162934

**Published:** 2016-09-08

**Authors:** Lucianna Vaccaro, Fernando Izquierdo, Angela Magnet, Carolina Hurtado, Mireya B. Salinas, Thiago Santos Gomes, Santiago Angulo, Santiago Salso, Jesús Pelaez, Maria Isabel Tejeda, Almudena Alhambra, Carmen Gómez, Ana Enríquez, Eva Estirado, Soledad Fenoy, Carmen del Aguila

The fifth author’s middle initial is incorrect. The correct name is Mireya B. Salinas. The correct citation is: Vaccaro L, Izquierdo F, Magnet A, Hurtado C, Salinas MB, Gomes TS, et al. (2016) First Case of Legionnaire's Disease Caused by Legionella anisa in Spain and the Limitations on the Diagnosis of Legionella non-pneumophila Infections. PLoS ONE 11(7): e0159726. doi:10.1371/journal.pone.0159726
