# Supplementary figures and images for: First Case of Legionnaire's Disease Caused by Legionella anisa in Spain and the Limitations on the Diagnosis of Legionella non-pneumophila Infections
Source: PLoS One. 2016 Jul 21;11(7):e0159726. doi: 10.1371/journal.pone.0159726 (PMC4956277; doi:10.1371/journal.pone.0159726)

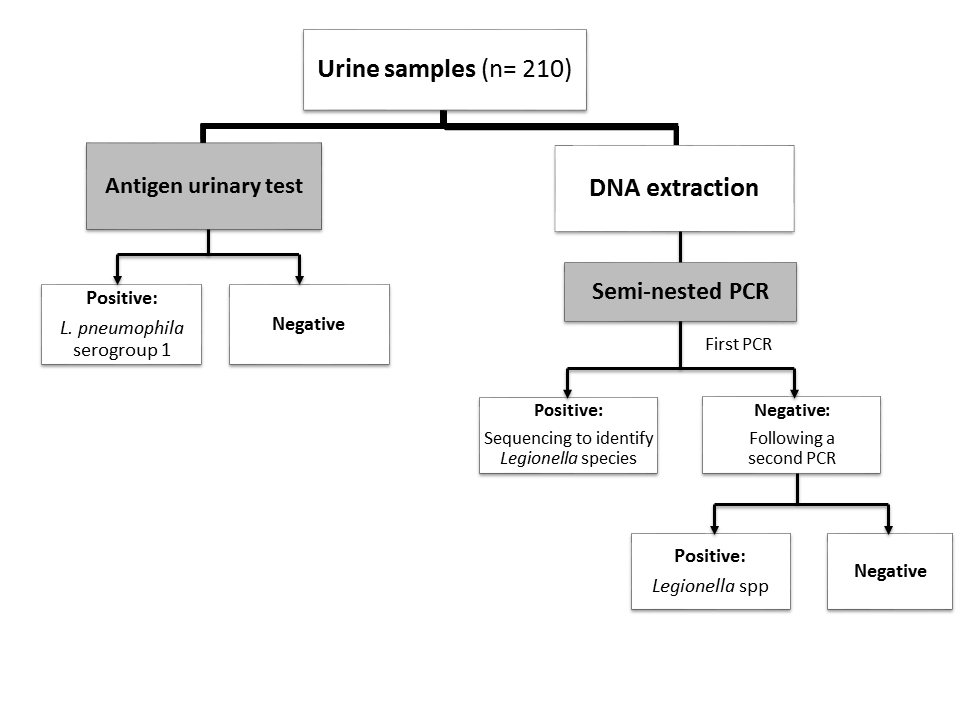

Supplement: S1 Fig — (TIF) [file pone.0159726.s001.tif]
