# Supplementary material for: First Case of Legionnaire's Disease Caused by Legionella anisa in Spain and the Limitations on the Diagnosis of Legionella non-pneumophila Infections
Source: PLoS One. 2016 Jul 21;11(7):e0159726. doi: 10.1371/journal.pone.0159726 (PMC4956277; doi:10.1371/journal.pone.0159726)
Supplement: S1 Table — (PDF) [file pone.0159726.s002.pdf]

S1 Table. The largest outbreaks of Legionnaires' disease from 1980 to 2015 in Spain.

Year	City	Province	Nº cases*	Fatalities (%)	Source of infection
1973	Benidorm	Alicante	89	3.3	Potable water system [1, 2]
1983	Zaragoza	Zaragoza	81	7.4	Hot water system [3]
1988	Barcelona	Barcelona	56	12.5	Unknown [4]
1991	Almuñecar	Granada	91	2.2	Cooling towers [5]
1996	Alcalá de Henares	Madrid	224	4.0	Cooling towers and drinking water reservoirs [6]
1999-2000	Alcoy	Alicante	177	6.2	Cooling towers [7]
2000	Barcelona	Barcelona	54	4.0	Cooling towers [8]
2001	Murcia	Murcia	449	1.1	Cooling towers [9]
2002	Mataró	Barcelona	151	1.4	Cooling towers [10]
2006	Pamplona	Navarra	146	0.0	Cooling towers [11]
2015	Manzanares	Ciudad Real	237	1.7	Decorative fountain [12]

*Numbers of confirmed cases of legionellosis.
The largest outbreaks of Legionnaires' disease in Spain were considered from 50 cases (n* > 50 cases).

References

1. Grist NR, Reid D, Najera R. Legionnaires' disease and the traveller. Ann Intern Med. 1979;90(4): 563-4. PubMed PMID: 434635.
2. Edelstein PH. Legionnaires´ Disease: History and Clinical Findings. In: Heuner K, Swanson M, editors. *Legionella*: molecular microbiology. Norfolk: Caister Academic Press; 2008. pp. 1-14.
3. Martínez Pérez E, Ocaña Cazalilla C, Cobos López J. Legionellosis: Características clínico-epidemiológicas y medidas sanitarias en el brote de Castillejos. Med Mil. 1984;40(2): 111-21.
4. Cayla JA, Sala MR, Plasencia A, Beneyto V, Sureda V, Llorens M, et al. Brote comunitario de enfermedad de los legionarios en Barcelona: investigación epidemiológica y medioambiental. Med Clin (Barc). 1989;93(14): 526-30. PubMed PMID: 2622252.
5. Centro Nacional de Epidemiología. Brote epidémico de neumonía por *Legionella* en Almuñecar. In: Boletín Microbiológico Semanal (BMS/48). Madrid: Instituto de Salud Carlos III; 1991. pp 2-4.
6. Centro Nacional de Epidemiología- Instituto de Salud Carlos III. Informe del brote de neumonía por *Legionella* de Alcalá de Henares. Madrid, abril 1997 (I). Bol Epidemiol Sem. 1997;5(14): 133-44. Available: http://gesdoc.isciii.es/gesdoccontroller?action=download&id=25/04/2013-f1837a2bac
7. Fernandez JA, Lopez P, Orozco D, Merino J. Clinical study of an outbreak of Legionnaire's disease in Alcoy, Southeastern Spain. Eur J Clin Microbiol Infect Dis. 2002;21(10): 729-35. doi: 10.1007/s10096-002-0819-9. PubMed PMID: 12415472.
8. Jerico Alba C, Nogues Solan X, Santos Martinez MJ, Felez Flor M, Garces Jarque JM, Marinosa Marre M, et al. Brote epidémico de neumonía comunitaria por *Legionella pneumophila* en Barcelona: "el brote de la Barceloneta". Efecto del diagnóstico y tratamiento precoz. Rev Clin Esp. 2004;204(2): 70-4. PubMed PMID: 15023304.
9. Garcia-Fulgueiras A, Navarro C, Fenoll D, Garcia J, Gonzalez-Diego P, Jimenez-Bunuales T, et al. Legionnaires' disease outbreak in Murcia, Spain. Emerg Infect Dis. 2003;9(8): 915-21. doi: 10.3201/eid0908.030337. PubMed PMID: 12967487.
10. Barrufet-Barque MP, Sauca-Subias G, Force-Sanmartin L, Felip-Benach A, Martinez-Perez E, Capdevila-Morell JA. Estudio de un brote de infección por *Legionella pneumophila*. Med Clin (Barc). 2006;126(5): 178-82. PubMed PMID: 16570380.
11. Castilla J, Barricarte A, Aldaz J, Garcia Cenoz M, Ferrer T, Pelaz C, et al. A large Legionnaires' disease outbreak in Pamplona, Spain: early detection, rapid control and no case fatality. Epidemiol Infect. 2008;136(6): 823-32. doi: 10.1017/S0950268807009077. PubMed PMID: 17662166.
12. *Legionella*. Brote en Manzanares-Cronograma de actuaciones. 2016 Feb [cited 25 Feb 2016]. In: Servicio de Salud de Castilla-La Mancha web [Internet]. Toledo: SESCAM. Available: http://sescam.castillalamancha.es/ciudadanos/legionela/brote-manzanares/cronograma-de-actuaciones
