# Supplementary material for: First Case of Legionnaire's Disease Caused by Legionella anisa in Spain and the Limitations on the Diagnosis of Legionella non-pneumophila Infections
Source: PLoS One. 2016 Jul 21;11(7):e0159726. doi: 10.1371/journal.pone.0159726 (PMC4956277; doi:10.1371/journal.pone.0159726)
Supplement: S2 Table — (PDF) [file pone.0159726.s003.pdf]

S2 Table. Cases of legionellosis attributed to *Legionella anisa* worldwide.

Year	Country	Case	Form of illness	Predisposing factors	Observations	Source of infection
1986	Australia	Sporadic case	LD	Diabetes mellitus, cancer	Characterization of a *L. anisa* strain isolated from a patient with pneumonia	Unknown [1]
1987	France	Sporadic case	LD	Cancer	First case reported worldwide	Hot water system (hospital) [2]
1988	USA	Outbreak	PF	Smoking (8%), chronic lung disease (3%), use of corticosteroids (3%)	First outbreak caused by *L. anisa*. 34 cases of PF were detected and 10 of 24 hotel employees presented positive serology for *L. anisa*	Decorative fountain (hotel) [3]
1989*	UK	Sporadic case	LD	Smoking	First case reported in the UK	Unknown [4]
1992-1993	USA	Serological survey	LD	Chronic obstructive pulmonary disease (43%), congestive heart failure (43%), asthma (21%), steroid use (14%), diabetes mellitus (14%)	4 cases of LD by *L. anisa* were detected from patients with CAP	Unknown [5]
2000*	France	Sporadic case	LD	Leukemia	*L. anisa* isolated from a sputum sample using an amoebal coculture method	Unknown [6]
2002	USA	Outbreak	PF	Smoking (9%), asthma (3%), immunodeficiency disorder (2%)	117 confirmed cases	Ornamental pool (restaurant) [7]
2009*	Japan	Sporadic case	-	Abdominal aortic aneurysm	Extrapulmonary infection (mycotic aortic aneurysm)	Unknown [8]
2013*	USA	Sporadic case	LD	Cancer	Extrapulmonary infection (patellar osteomyelitis)	Unknown [9]
2014	France	Sporadic case	LD	Asthma, diabetes mellitus, aortic valve replacement, pacemaker	Extrapulmonary infection (chronic endocarditis)	Unknown [10]

*Date of the article accepted for publication. In these cases, the date of infection is not known.
Legionnaires´ disease (LD); Pontiac Fever (PF); Community Acquired Pneumonia (CAP).

References

1. Thacker WL, Benson RF, Hawes L, Mayberry WR, Brenner DJ. Characterization of a *Legionella anisa* strain isolated from a patient with pneumonia. J Clin Microbiol. 1990;28(1):122-3. Epub 1990/01/01. PubMed PMID: 2405005.
2. Bornstein N, Mercatello A, Marmet D, Surgot M, Deveaux Y, Fleurette J. Pleural infection caused by *Legionella anisa*. J Clin Microbiol. 1989;27(9):2100-1. PubMed PMID: 2778073.
3. Fenstersheib MD, Miller M, Diggins C, Liska S, Detwiler L, Werner SB, et al. Outbreak of Pontiac fever due to *Legionella anisa*. Lancet. 1990;336(8706):35-7. Epub 1990/07/07. doi: 0140-6736(90)91532-F [pii]. PubMed PMID: 1973219.
4. Fallon RJ, Stack BH. Legionnaires' disease due to *Legionella anisa*. J Infect. 1990;20(3):227-9. Epub 1990/05/01. doi: 0163-4453(90)91144-3 [pii]. PubMed PMID: 2341733.
5. McNally C, Hackman B, Fields BS, Plouffe JF. Potential importance of *Legionella* species as etiologies in community acquired pneumonia (CAP). Diagn Microbiol Infect Dis. 2000;38(2):79-82. Epub 2000/10/18. doi: S0732-8893(00)00181-4 [pii]. PubMed PMID: 11035237.
6. La Scola B, Mezi L, Weiller PJ, Raoult D. Isolation of *Legionella anisa* using an amoebic coculture procedure. J Clin Microbiol. 2001;39(1):365-6. Epub 2001/01/04. doi: 10.1128/JCM.39.1.365-366.2001. PubMed PMID: 11136802.
7. Jones TF, Benson RF, Brown EW, Rowland JR, Crosier SC, Schaffner W. Epidemiologic investigation of a restaurant-associated outbreak of Pontiac fever. Clin Infect Dis. 2003;37(10):1292-7. Epub 2003/10/30. doi: CID31317 [pii]10.1086/379017. PubMed PMID: 14583861.
8. Tanabe M, Nakajima H, Nakamura A, Ito T, Nakamura M, Shimono T, et al. Mycotic aortic aneurysm associated with *Legionella anisa.* J Clin Microbiol. 2009;47(7):2340-3. Epub 2009/05/22. doi: 10.1128/JCM.00142-09JCM.00142-09 [pii]. PubMed PMID: 19458178.
9. Sanchez MC, Sebti R, Hassoun P, Mannion C, Goy AH, Feldman T, et al. Osteomyelitis of the patella caused by *Legionella anisa*. J Clin Microbiol. 2013;51(8):2791-3. Epub 2013/06/14. doi: 10.1128/JCM.03190-12JCM.03190-12 [pii]. PubMed PMID: 23761141.
10. Compain F, Bruneval P, Jarraud S, Perrot S, Aubert S, Napoly V, et al. Chronic endocarditis due to *Legionella anisa*: a first case difficult to diagnose. New microbes and new infections. 2015;8:113-5. doi: 10.1016/j.nmni.2015.10.003. PubMed PMID: 26693025.
